# Ill-Defined Problem Solving Does Not Benefit From Daytime Napping

**DOI:** 10.3389/fpsyg.2020.00559

**Published:** 2020-04-09

**Authors:** Małgorzata Hołda, Anna Głodek, Malwina Dankiewicz-Berger, Dagna Skrzypińska, Barbara Szmigielska

**Affiliations:** ^1^Section of Sleep Psychology, Institute of Psychology, Jagiellonian University, Cracow, Poland; ^2^Department of Educational Psychology, Institute of Psychology, Pedagogical University of Cracow, Cracow, Poland

**Keywords:** nap, sleep sleep/wake cognition, problem solving, ill-defined problems, divergent thinking, creative reasoning

## Abstract

The main goal of the present study was to explore the role of sleep in the process of ill-defined problem solving. The results of previous studies indicate that various cognitive processes are largely dependent on the quality and quantity of sleep. However, while sleep-related memory consolidation seems to be well-grounded, with regard to the impact of sleep on problem solving, existing research yields mixed and rather inconclusive results. Moreover, this effect has been mainly tested using simple and well-defined, common laboratory problems, such as the remote associate test (RAT), crossword and anagram puzzles, numeric and logic problems, etc. What is lacking is research on the effect of sleep on solving more complex and more real-life oriented ill-defined problems. In the present study, we hypothesized that sleep can improve performance in solving this kind of problems. The study involved 40 participants, randomly assigned to two experimental conditions: sleep group and waking group. The experimental protocol comprised three stages: problem presentation, retention interval, and testing stage. The problem was presented to the participants in the form of an interactive computer game concerning a complex, elaborate crime story. During the retention interval, the participants—depending on the condition—took a nap or stayed awake; sleeping participants underwent polysomnography recording, while waking participants performed activities not related to the experimental problem. In the testing stage, participants tried to solve the presented problem. The solutions generated were assessed both for quality (reasonableness, consistency, and story recall) and creativity (fluency, flexibility, originality, and elaboration). Contrary to expectations, we found no effect of sleep on ill-defined problem solving. Neither quality nor creativity of the solutions generated by the participants was higher in the nap group than in the waking group. There were also no performance improvements with regard to any sleep stage or incidence of dreams. Our study adds to a growing body of evidence that sleep probably might provide an incubation gap, but not a facilitating environment serving the purpose of problem solving, at least with regard to ill-defined problems.

## Introduction

The results of previous research indicate that sleep is essential for many cognitive processes. It has been repeatedly demonstrated that sleep loss or insufficient sleep is related to cognitive decline and can adversely affect a variety of cognitive and emotional abilities, including alertness, vigilance, and attention, emotional intelligence and stress management skills, memory and learning, fluid intelligence, executive functions, reasoning, and problem solving (e.g., Linde and Bergström, 1992; Fairclough and Graham, 1999; Drummond et al., 2000; Williamson and Feyer, 2000; Diekelmann et al., 2008; Killgore et al., 2008; Kronholm et al., 2009; Nebes et al., 2009; Johnston et al., 2010; Lim and Dinges, 2010; Frings, 2011; Horne and Moseley, 2011; Plessow et al., 2011; Xu et al., 2011; Jackson et al., 2013).

Sleep appears to be critical particularly for memory consolidation—a growing body of evidence shows that sleep contributes to stabilization of information acquired before sleep (Gais and Born, 2004; Stickgold and Walker, 2005; Diekelmann and Born, 2010; Conte and Ficca, 2013; Rasch and Born, 2013). This effect was found both for procedural and declarative memory (Stickgold et al., 2000; Mednick et al., 2002, 2003; Walker et al., 2003; Alger et al., 2010; Wamsley et al., 2010b; Antony et al., 2012; Payne et al., 2012; Cousins et al., 2019), and it is believed to be a result of reactivation and stabilization of recently encoded memory representations in different sleep stages (Gais and Born, 2004; Diekelmann and Born, 2010; Lu and Göder, 2012; Antonenko et al., 2013; Rasch and Born, 2013; Llewellyn and Hobson, 2015).

However, the effect of sleep seems to go beyond only a simple replay of memories. There is accumulating evidence suggesting that sleep-related memory consolidation helps to reorganize and integrate memories with preexisting knowledge, which may enhance the abstraction of underlying rules and associations. Thereby, complex cognitive processes, such as reasoning, insight, problem solving, and creativity may benefit as well (Llewellyn, 2016; Chambers, 2017). As demonstrated by Lau et al. (2011), sleep enhances the reorganization of discrete memory traces into flexible relational memory networks. Djonlagic et al. (2009) found a sleep-dependent improvement in categorizing objects, and Nieuwenhuis et al. (2013), using an artificial grammar learning paradigm, reported that classification performance improved after sleep. It was also demonstrated that sleep facilitates generation of false memories in the Deese, Roediger, and McDermott (DRM) false memory paradigm (Diekelmann et al., 2010). These findings suggest that sleep plays a critical role in integrating memories, extracting rules, creating connections, and semantic generalization of newly encoded information.

Sleep was also demonstrated to inspire insight and enhance problem solving. In the study conducted by Wagner et al. (2004) with the number reduction task (NRT), more than twice as many subjects gained insight into the hidden rule after a night of sleep as after a respective period of wakefulness. Sleep-related insight was confirmed in numerous studies (e.g., Yordanova et al., 2010; Yordanova et al., 2012; Debarnot et al., 2017). Similar results were obtained by Beijamini et al. (2014), who found that, after a nap, subjects were almost twice as likely than after waking interval to solve a video game problem involving logical reasoning. Monaghan et al. (2015) exploited analogical problems, which require applying a known solution from one problem to a related problem, and they showed that sleep facilitated such analogical transfer mainly due to structural generalization across problems. In another study (Sio et al., 2013), using a set of remote-associate tasks (RAT) varying in difficulty, sleep enhanced solving difficult problems, while there was no effect for easy problems. In line with these findings, sleep turned out to be beneficial to creativity assessed with classical measures like the abbreviated torrance test for adults (Drago et al., 2011) or the unusual uses task (Ritter et al., 2012).

These benefits of sleep are mostly connected with slow-wave sleep (SWS; Yordanova et al., 2010, 2012; Beijamini et al., 2014) and rapid eye movement (REM) sleep (Walker et al., 2002; Cai et al., 2009; Djonlagic et al., 2009; Sterpenich et al., 2014); although Drago et al. (2011) found correlations of creativity not only with stage 4 but also with stage 1 of non-REM (NREM) sleep. Similarly as in sleep-dependent learning, the mechanism of the facilitating effect of sleep on reasoning, creativity, and problem solving is believed to be the neuronal memory reprocessing during sleep, including reactivation, integration, and restructuration of new memory representations (Wagner et al., 2004; Yordanova et al., 2010, 2012; Chambers, 2017). According to the information overlap to abstract (iOtA) model proposed by Lewis and Durrant (2011), cognitive abstraction is based on an overlapping replay of newly encoded memories during slow-wave sleep, which leads to the integration of newly learned information into existing cognitive schemata, as well as to the abstraction of the gist, and thus to the formation of new schemata. Likewise, Lewis et al. (2018) propose that abstracting rules from corpuses of learned information is possible owing to memory replay mechanisms in non-REM sleep, while novel associations may be formed as a result of replay in REM sleep. Thus, it is the iterative interleaving of REM and non-REM sleep across a night that is thought to boost the formation of complex knowledge frameworks. This mechanism allows to recombine and restructure memories, facilitating creative thinking.

A number of studies suggest also a possible relationship between cognition and dreaming. According to Payne and Nadel (2004), dreaming reflects long-term memory consolidation, which strengthens the neural traces of recent events, integrates new traces with existing memories and prior knowledge, and sustains their stability. In a study by Wamsley et al. (2010a), improved performance at retest in a virtual navigation task was indeed strongly associated with task-related dream content during an intervening afternoon nap, while task-related thoughts during respective period of wakefulness did not yield any improvement. These findings support the view that dream experiences reflect the offline reactivation of recently formed memories during sleep. In line with this model, Fogel et al. (2018) demonstrated that the extent to which some novel experiences are learned is related to the extent to which these experiences are incorporated into the dream content, while the extent of this incorporation is related to interindividual differences in cognitive abilities. Dreams are also thought to inspire creativity and problem solving (Barrett, 2001a, b; Llewellyn, 2016). For instance, it turned out that musicians dream about music more often than non-musicians and that the music from their dreams is often novel and original (Uga et al., 2006). Likewise, film makers more often than the general population report that dreams affect their creative activity (Pagel et al., 1999). In two questionnaire studies involving ordinary people, the majority of participants reported experiencing sleep- or dream-related insights occasionally or regularly, and many admitted that these stimulating dreams played a considerable role in their lives (Schredl and Erlacher, 2007; Perdomo et al., 2018). In another study (Barrett, 1993), participants were instructed to incubate dreams addressing problems of their own choice. About half of them recalled a dream that they judged as related to their problem; moreover, a majority of these dreams were believed to contain a problem solution.

Nevertheless, there are also some contradictory evidence, undermining the effect of sleep on problem solving. In one of the first laboratory studies on this issue, Cartwright (1974) did not find any improvements in the performance on intellectual tasks after sleep compared to wakefulness. In another study, Landmann et al. (2016) used the compound remote associate (CRA) task, which is a verbal creativity task, and reported sleep-related improvements in strengthening, but not in the creative reorganization, of newly encoded memories. As reported by Debarnot et al. (2017), sleep facilitates insight in problem solving only in young adults, while in old adults, no sleep-dependent improvement in problem solving was observed. Two recent studies corroborated these results. Schönauer et al. (2018) found no effect of sleep on the solution of classical insight problems or magic tricks. Neither general solution rates nor the number of solutions accompanied by sudden subjective insight were influenced by a nap compared to waking period, and no significant correlations between performance and the time spent in specific sleep stages were obtained. This findings were supported in another study by Brodt et al. (2018), who demonstrated that an incubation period positively affected solution rates in classical riddles; however, spending the period of incubation asleep yield no additional benefit. These results suggest that sleep might not be facilitating for problem solving in general, or for solving particular problems, and that at least some of the sleep-related improvements in complex cognitive processes might be a result of incubation rather than sleep itself.

To summarize, although there is a growing body of research on the impact of sleep on problem solving, their results are still rather inconclusive. Moreover, this effect has been mainly tested using simple and well-defined, common laboratory problems, such as the RAT and analogical problems (Cartwright, 1974; Cai et al., 2009; Sio et al., 2013; Monaghan et al., 2015), crossword and anagram puzzles (Cartwright, 1974; Walker et al., 2002; Brodt et al., 2018), numeric and logic problems (Wagner et al., 2004; Yordanova et al., 2010; Beijamini et al., 2014; Brodt et al., 2018), or standard paper-and-pencil tests (Cartwright, 1974; Drago et al., 2011; Ritter et al., 2012). This kind of clearly structured problems are off course easily brought into the psychological laboratory. However, there is some concern that such tasks may not appropriately capture real-world cognitive functioning and problem solving, since problems frequently encountered in the real world, such as political, economic, science, societal, moral, or personal problems, as well as daily life problems, are often much more complex and mainly ill structured. What is lacking thus is research on the effect of sleep on solving more complex and more real-life oriented ill-defined problems which feature open boundaries and have no well-determined solutions. Along with life-long learning and collaboration skills, solving complex, ill-defined problems is believed to be one of the most important competencies in the modern world, and it is particularly important to study how people deal with complex, dynamic, and uncertain real-world challenges (Jonassen, 2000; Greiff et al., 2014; Shute et al., 2016; Dörner and Funke, 2017). Given that well- and ill-defined problem solving are independent to a large extent and require separate cognitive processes (Schraw et al., 1995; Welter et al., 2017), we can suppose that the effect of sleep might be different in these two types of problem situations.

Well-defined (well-structured) problems are those that contain a clear specification of three elements of the problem space: the initial state (the problem situation), the set of operators (rules and strategies) to solve the problem, and the goal state (the solution). Ill-defined problems lack all or most of the information required to reach a solution, i.e., they leave at least one of the three elements (initial state, solution operators, or goal state) not clearly specified (Reitman, 1965; Newell and Simon, 1972; Eysenck and Keane, 2000). Well-defined problems tend to have a single, convergent, absolutely correct, and knowable solution, while ill-defined problems often, apart from offering incomplete, ambiguous, open to interpretation, or uncertain initial states and sets of operators, may be solved with a multitude of potentially effective solutions (Kitchener, 1983; Simon, 1986; Moreau and Engeset, 2016). Solving an ill-defined problem often involves exploration and experimentation along with developing, evaluating, and selecting a solution from a set of multiple ideas generated in the course of the problem solving process (Guilford, 1967/1978; Dörner and Funke, 2017), and the cognitive abilities required for ill-defined problem solving are comparable to those required for creativity tasks (Welter et al., 2017). Solving an ill-defined problem can be thus considered as an act of creative thinking (Moreau and Engeset, 2016). Nevertheless, creativity or divergent thinking is not sufficient to solve ill-defined problem. The solving process must be eventually brought to an end—the set of generated possible solutions must be narrowed, and each of the solutions has to be valued with respect to their quality and functionality in the context for which they were intended. This involves also convergent thinking, and thus, both convergent and divergent thinking processes intertwine and cooperate to reach a viable solution of an ill-defined problem in a process called creative reasoning: Divergent thinking is responsible for creating new ideas, while convergent thinking ensures correct and logical assessments and choices (Cropley, 2006; Jaarsveld et al., 2010). Therefore, when investigating ill-defined problem solving, it seems reasonable to take into account both efficacy, or quality, and creativity of the solution.

The main goal of the present study was to test the possible effect of sleep on the process of ill-defined problem solving. In line with some previous findings, we hypothesized that sleep can improve performance in solving ill-defined problems. In the present study, a nap paradigm was adopted. It has been demonstrated repeatedly that a short daytime nap may yield similar memory or reasoning improvement as an overnight sleep. Concurrently, it allows to avoid confounds by sleep–wake cycle and sleep deprivation of participants (Mednick et al., 2003; Lahl et al., 2008; Beijamini et al., 2014; Payne et al., 2015). In the present study, the experimental protocol comprised three stages. First, participants were acquainted with a complex, ill-defined problem, which they tried to solve after a retention interval filled with sleep (90 min nap) or wakefulness. We expected that nap participants would solve the problem more efficiently and more creatively. We also explored if dreams could benefit problem solving, i.e., if participants’ performance is related to the incidence of dreams or dream content.

## Materials and Methods

To test the feasibility of methods and procedures, a pilot study was conducted first; then, some corrections were applied to the main study protocol and research tools. Subsequently, the main study was conducted. Owing to the changes introduced to the study procedure, it was not possible to include the results of the pilot study in the final analyses. However, some preliminary analyses of the mere pilot study findings were also conducted. Detailed results of the pilot study followed by the modifications of the study protocol implemented after the pilot study are presented in Supplementary Material.

### Participants

Participants were recruited by internet advertisements and then qualified to the study on the basis of the screening test. Exclusion criteria were neurological or psychiatric disorders, the use of sleep-affecting or nervous-system-stimulating drugs, and inability to sleep during the day. Overall, 134 individuals filled out the screening test, and 88 individuals who met the inclusion criteria were invited to take part in the study. Eventually, 40 participants came forward and finished all the steps of the study, 31 female and 9 male, aged 19–35 (mean age, 23.3). Participants were students of various programs or had higher education (16 participants were studying psychology or had a psychology degree). They were financially compensated for their participation. Additionally, 20 participants took part in a pilot study (see Supplementary Material).

The participants were randomly assigned to two experimental conditions: sleep group and waking group. One sleep participant was excluded due to some abnormalities in the PSG recording and suspicion of sleep disorder, leaving a total of 39 participants for analysis (19 in the sleep group, 14 female and 5 male; 20 in the waking group, 16 female, 4 male).

### Procedure and Measures

#### Screening

The screening test, used during the recruitment process, included questions concerning main exclusion criteria (neurological or psychiatric disorders, the use of sleep-affecting or nervous-system-stimulating drugs, and inability to sleep during the day). Additionally, it covered basic demographic and health variables, sleep patterns and sleep quality, as well as experience with computer games and crime stories and riddles (books, films, etc.).

One week before the experimental day, participants selected for the study were invited to the laboratory to take the APIS-Z battery (Ciechanowicz et al., 1995)—a multidimensional standardized paper-and-pencil test commonly used to assess general intelligence. APIS-Z is designed especially to assess intelligence in students and persons with higher education. It comprises of eight tests, measuring four types of cognitive abilities: abstract–logical, verbal, visuospatial, and social abilities. It has high internal consistency for the total score and satisfactory stability and validity. In the present study, only the total IQ score was taken into account to control the influence of this variable on participants’ performance. Participants were also informed in detail about all the experimental procedures and study goals and instructed to abstain from caffeine and sleep-affecting drugs directly before the study. Then, they were asked to keep sleep logs for a week before the study to monitor their sleep–wake cycle. The following variables were considered for further analysis: average sleep time, average sleep onset and average wake-up time, sleep onset the night before the experiment, wake-up time on the day of the experiment, and sleep time the night previous to the experiment.

#### Experimental Protocol

**FIGURE 1:**
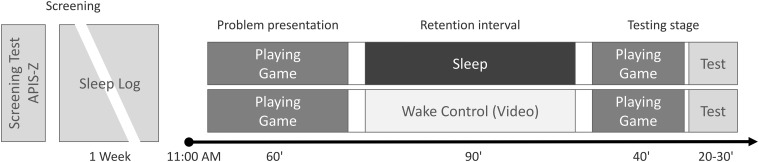
Study protocol. One week before the experimental day, participants filled out the questionnaires (screening test and APIS-Z battery) and started sleep log. On the experimental day, they were acquainted with the problem in the form of a computer game first; then, they took a nap (sleep group) or watched videos (waking group). In the testing stage, participants finished the game and tried to solve the problem (filling out the final test).

Following the screening process, participants took part in the main, experimental part of the study. Upon arrival at the laboratory at 11:00 AM, participants were informed in detail about all the experimental procedures and instructions and randomly assigned to experimental condition. The experimental protocol comprised three stages: problem presentation, retention interval, and testing stage. The problem was presented to the participants in the form of an interactive computer game concerning a complex, elaborate crime riddle. Participants played the game for 60 min. During the retention interval, the participants—depending on the condition—took a 90-min nap or stayed awake. Sleeping participants underwent polysomnography recording, while waking participants performed activities not related to the experimental problem (they watched nature documentary videos). In the testing stage, all participants played the game for another 40 min and then took the final test, comprising questions concerning the presented problem. The experimental design is schematically presented in Figure 1.

##### Behavioral task

The experimental problem was presented to participants in the form of the interactive video game *Her story*.^1^ As demonstrated, using computer games not only allows to create a situation of more interactive nature, which may facilitate motivation to struggle with the experimental problem (Przybylski et al., 2010) but also allows to reconcile designing complex and real-world problem situations with methodological requirements of laboratory experiments (Wouters et al., 2013).

In the game used in the present study, the player searches and sorts through a database of video clips from fictional archived police interviews and uses the clips to solve the case of a murder. The interviews are unable to be watched in their entirety or in proper, chronological order; only fragmented short clips are available. Furthermore, merely answers of the interrogated person can be heard, while the questions of the off-screen detective remain unknown. The player takes on the role of the person sitting before a police computer terminal, attempting to solve the case by piecing together information, like in a real police investigation; and like in a real investigation, the objective is uncertain—the player gets only vague instruction to “resolve the case” and does not exactly know what is the overriding goal and successive steps to take. Moreover, the game does not have a classical end (“win/lose”) and a definitive solution, so the whole story might be variously explored and interpreted.

Participants were not provided with any background information before playing. The instruction was as follows: “*You will play a computer game now. Your task is to solve a complex problem, a sort of criminal case. You will play the game for an hour.*” If participants asked for more information, both before and during playing the game, they were only instructed: “*try to acquire as much information as you can, to resolve the case*,” and if they kept asking, they were informed: “*it is your task to find the solution on your own, so the experimenter cannot advise anything*.” The game mechanics is very simple, and it resembles the google search engine—the player just thinks up keywords and types them in the browser pane; then, the database returns clips where the interrogated person speaks those words. In the beginning of the game, the player sees an old-school computer desktop with the database activated, and the word “murder” entered in the browser pane. When the player clicks “search,” first few clips can be seen. Then, after watching those first clips, the player can search the database using other words that he/she expects to bring some more information about the case. There are almost 300 clips—shorter or longer, and including key information for the case or just less important, marginal threads—therefore, the player has to carefully choose the keywords to get to the most informative and interesting clips. Some screenshots and videos from the game are available from the producer’s website.

This game was chosen due to its non-linear storytelling, open to interpretation and fragmented narrative, uncertain goal state and vague means of achieving it, as well as ambiguity and the lack of a definitive solution. Solving the problem presented in the game requires exploring and analyzing a large amount of information including conflicting assumptions and evidence, identifying problems, and planning of successive steps. Because the story is complex and multithreaded, the problem definition must be changed dynamically as the player discovers subsequent facts. This kind of problem fits the definition of ill-defined problem (Reitman, 1965; Newell and Simon, 1972; Kitchener, 1983; Simon, 1986; Eysenck and Keane, 2000). Given that complex problem solving is often dependent on prior knowledge and on emotional and motivational processes (Dörner and Funke, 2017), a crime riddle was chosen because it seems to be quite common and universal, as well as interesting, attracting, and motivating for participants, due to its interactive form and intriguing plot.

##### Polysomnography recording

In-laboratory sleep recordings were performed in accordance with standardized techniques, using a Comet PSG system (Grass Technologies). Electroencephalogram (EEG) (from scalp locations: F3, F4, C3, C4, O1, and O2, according to the 10–20 system), electrooculogram (EOG) (from the left and right outer canthi of the eye), and electromyogram (EMG) (from the chin muscles) were recorded with gold-plated cup electrodes applied to the skin. EEG and EOG channels were referenced to the contralateral mastoids (M1 and M2); the EMG channel was recorded as a bipolar derivation. The ground electrode was placed on the forehead (Fpz). Electrode impedances were lower than 5 kΩ. All participants from the sleep group were permitted a 90-min opportunity within the retention interval to attempt napping, and after 90 min, they were awakened regardless of the sleep stage they were in. After the nap, participants were also asked if they had any dreams.

Sleep stages were visually scored in 30-s epochs by a single expert in accordance with the Manual for Scoring from American Academy of Sleep Medicine (Berry et al., 2015) using TWin software (Grass Technologies). Scoring was performed blind to participants’ behavioral task performance. The following variables were taken into account for further analysis: total sleep time, particular sleep stages time (N1, N2, SWS, and REM), sleep latency, wake after sleep onset (WASO), and the number of dreams. Dream content was analyzed with regard to the incidence of incorporations of the presented problem by a single expert blind to participants’ behavioral task performance.

##### Testing stage

In the testing stage, participants tried to solve the presented problem. First, they played the game and could explore the problem for another 40 min. Then, they took the final test. Because the problem presented in the game does not have one specified solution and the game does not have a classical end (“win/lose”), it is not possible to apply usual “correct/false” indicator to assess if the participant solved the problem or not. Therefore, a more complex procedure was used to measure participants’ performance, consisting in a paper-and-pencil test scored by a panel of expert raters. The test was prepared particularly for the purpose of the present study and revised after the pilot study (see Supplementary Material for details)—vague or suggesting questions were modified or removed, and some questions were added or expanded to allow obtaining more elaborate and detailed responses, which might more clearly reveal participants’ reasoning process. New scoring rules were also prepared.

Finally, the test included 38 open questions concerning both the facts from the game (18 questions, e.g., “Did Simon have an affair?,” “Where was Simon’s body found?,” “Who got a mirror from Simon?,” “What alibi did the interrogated woman present?” etc.) and participants’ conclusions and interpretations of the story (20 questions, “When and why did the interrogated woman change her testimony?,” “Who killed Simon? Why was he killed?,” “What role did a mirror play in the whole story?,” “Do you think that the interrogated woman lied? When?,” “Which threads of the story do you consider most important in the view of the investigation?,” “What might have happened after the last interrogation? What might be the next step of the police in the case of Simon’s murder?” etc.). Time for filling out the test was not limited; it usually took approximately 20–30 min.

Four independent expert raters, blind to participants’ group assignment, assessed the solutions generated by the participants for their quality (how effectively the participant solved the problem) and creativity (how creative the solution was). Quality of the solution was assessed with respect to three criteria: reasonableness, consistency, and story recall. Creativity of the solution was assessed with respect to four classical criteria, proposed by Guilford (1967/1978) and Torrance (1974): fluency, flexibility, originality, and elaboration. Scores for each of the criteria were summed for each rater and then averaged.

*Reasonableness* refers to validity and pertinence of the solution. Although the presented problem does not have a simple solution and the story might be variously interpreted, careful investigation of all the facts shows that some explanations are more and some are less probable and justified. The reasonableness scale measures the convergence of participants’ interpretations with this most probable solution. Participants’ answers were scored with 0 points (invalid or no answer), 1 point (valid, but not profound and insightful answer), or 2 points (valid and profound, insightful answer, logical and well-grounded in the context of the whole story, not only a single thread or situation). All the questions were included in this score, and the scores for each answer were summed up; therefore, the minimum score for this scale was 0, and the maximum was 76 points (the more points, the more reasonable the solution was).

*Consistency* is a measure of the coherence of the solution, i.e., consistency of responses to different questions. All the questions were included in this score, and the raters assessed if the answers compose a logical plot—first, the raters read answers to three key questions provided by the participant to initially qualify his/her interpretation of the story, and then, they assessed each answer with respect to its consistency with the participant’s interpretation. Participants’ answers were scored with 0 points (no answer), C (answer consistent with the participant’s interpretation), P (answer only partly consistent with the participant’s interpretation), or I (answer inconsistent with the participant’s interpretation). The result in this scale was the ratio of the consistent answers to all answers (all responded questions): (C + 0.5 P)/(C + P + I). This kind of index was used here instead of the sum to avoid the missing-responses bias (participants with many missing responses, i.e., those who answered only few questions but all their answers were consistent would have lower scores than participants who answered all the questions but in an inconsistent way). The minimum score for this scale was 0 points, and the maximum was 1 point (the closer the score to 1, the more consistent the solution was).

*Story recall* refers to the number of properly recalled facts from the game. Unlike the reasonableness score, in this scale, participants’ answers were assessed with respect to the basic knowledge of isolated facts, not the whole picture of the plot. However, due to the task specificity, probably not all participants acquired all the facts because they had not watched all the key clips. Therefore, this score plausibly depends also on participants’ ability to effectively search and sort information and to solve the problem, being not exclusively a memory indicator. It is partly a measure of the amount of information participants have reached to, not the amount of information they have recalled from the information they were presented. The answers were scored with 0 points (incorrect or no answer), 1 point (correct, but perfunctory, not detailed answer), or 2 points (correct and detailed answer). Four questions impossible to respond unequivocally on the basis of the game (questions concerning some additional interpretations, further course of events, etc.) were excluded from this score. The scores for each answer were summed up; thus, the minimum score for this scale was 0, and the maximum was 68 points (the more points, the better recall).

*Fluency* refers to the number of solutions. Participants’ answers were scored with 0 points (incorrect or no answer), 1 point (any relatively correct, single answer), or 2 points (two or more probable and anyhow justified explanations). All the questions were included in this score, and the scores for each answer were summed up; thus, the minimum score for this scale was 0, and the maximum was 76 points (the more points, the more fluent the solution was).

*Flexibility* is a measure of the diversity of solutions. If an answer included more than one probable and justified explanation, these explanations were assessed with respect to their similarity. Each answer was scored with 0 points (single or no answer), 1 point (two or more similar explanations), or 2 points (two or more different explanations). Four questions that may have been responded only in one way were excluded from this score. The scores for each answer were summed up; therefore, the minimum score for this scale was 0, and the maximum was 68 points (the more points, the more flexible the solution was).

*Originality* refers to the rarity and unusualness of the solution. Participants’ answers were scored with 0 points (typical answer) or 1 point (rare, original answer, submitted by only one or two participants; all original responses were taken into account; therefore, if more than one original response for one question was submitted, more than one point was scored). All the questions were included in this score, and the scores for each answer were summed up, the minimum score for this scale was 0, and the maximum theoretically was not limited due to the fact that participants could get more than one point for each answer (the more points, the more original the solution was).

*Elaboration* refers to the effort put in developing the solution, i.e., the number of words or details in the description, regardless of its correctness. The answers were scored with 0 points (no answer), 1 point (short, single answer), 2 points (longer, more elaborate answer including some additional details), or 3 points (exhaustive, comprehensive description). All the questions were included in this score, and the scores for each answer were summed up; therefore, the minimum score for this scale was 0, and the maximum was 114 points (the more points, the more elaborate the solution was).

### Statistical Analyses

Kendall’s coefficient of concordance (*W*) was performed to measure agreement among the four raters who scored the problem solutions generated by the participants in the final test. To test the effect of sleep on participants’ performance, independent *t*-tests were used, adjusted for multiple comparisons with the sequentially rejective multiple-test procedure (Bonferroni–Holm correction; Holm, 1979). Some additional independent *t*-tests and *χ*^2^ together with regression analysis were used to assess possible relationships with other factors.

## Results

To test the agreement among the four raters who assessed participants’ solutions, Kendall’s coefficient of concordance was calculated. The obtained coefficients were high for reasonableness, story recall, fluency, flexibility, originality, and elaboration, and lower, but still acceptable, for consistency (see Table 1). Therefore, the raters’ scores were averaged, and those aggregated scores were used in further analyses.

**TABLE 1:** Coefficients of concordance among the four raters’ scores of participants’ solutions.

	**Kendall’s *W***
Reasonableness	0.96
Consistency	0.42
Story recall	0.98
Fluency	0.96
Flexibility	0.91
Originality	0.79
Elaboration	0.93

**FIGURE 2:**
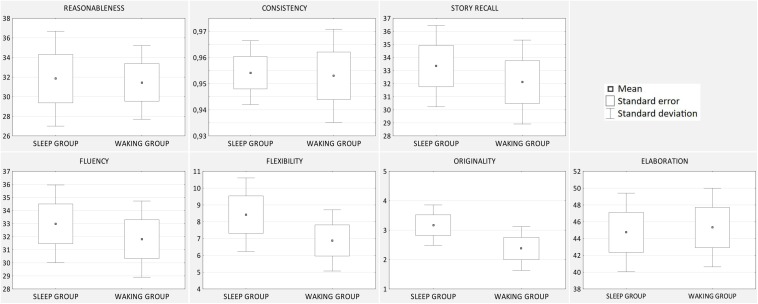
Effects of sleep on problem solving. Differences between sleep and waking group in task performance (means, standard errors, and standard deviations).

To test the effect of sleep on problem solving a number of pairwise comparisons (*t*-tests for independent samples, adjusted for multiple comparisons) was conducted to compare the performance of sleep and waking participants. None of the effects was significant. The results are presented in Table 2, and Figure 2 shows box-and-whisker plots for all the effects. There was also no effect with regard to any sleep stage or incidence of dreams. The nap architecture, obtained by PSG, is shown in Table 3. All participants from the sleep group actually fell asleep, with the shortest nap lasting for 10.5 min and the longest for 82 min; 16 participants had achieved slow-wave sleep, and 7 had achieved REM sleep. Moreover, 13 participants recalled dreams, although their content was mostly very short and undetailed, and none of the dreams seemed to be related to the experimental problem.

**TABLE 2:** Effects of sleep on problem solving.

	**Sleep group (*N* = 19)**		**Waking group (*N* = 20)**		***t*(37)**	***p***
		
	**Mean**	***SD***	**Mean**	***SD***		
Reasonableness	31.8	10.76	31.5	8.57	0.122	0.903
Consistency	0.95	0.03	0.95	0.04	0.109	0.914
Story recall	33.3	6.90	32.1	7.32	0.534	0.597
Fluency	33.0	6.60	31.8	6.65	0.553	0.584
Flexibility	8.4	4.86	6.9	4.15	1.062	0.295
Originality	3.2	1.53	2.4	1.70	1.538	0.133
Elaboration	44.7	10.39	45.3	10.68	–0.170	0.866

**TABLE 3:** Nap architecture.

	**Sleep duration (minutes)**				***N***
		
	**Mean**	***SD***	**Min**	**Max**	
TST	53.08	23.16	10.50	82.00	19
N1	13.42	6.23	3.00	29.00	19
N2	22.39	11.49	4.00	42.00	18
SWS	16.81	17.50	0.50	53.00	16
REM	11.64	2.76	7.00	14.50	7
WASO	22.92	19.97	2.50	71.50	19
Sleep latency	14.03	10.70	2.50	36.50	19
Number of dreams	1.15	0.38	1.00	2.00	13

To explore any confounding factors related to participants’ performance, the demographic data and sleep patterns were analyzed. Sleep and waking groups were balanced for age, sex, IQ, and education (the distribution of participants who were studying psychology or had a psychology degree was similar between groups). There was also no difference between groups in any sleep-pattern variable (both obtained from the Screening Test and Sleep Logs) and experience with games or crime riddles (see Table 4). To investigate other possible factors related to the problem-solving process, some additional analyses were conducted. Multiple regression, depicted in Table 5, indicated that IQ and sex were strongly related to participants’ performance: IQ was related to story recall, reasonableness, and fluency (the higher the IQ score, the higher the problem-solving scores), while sex was related to story recall, fluency, flexibility, originality, and elaboration (women had higher scores than men). These two variables explained for ∼40% of the variance in story recall and fluency. There was no effect for education (psychological vs. non-psychological).

**TABLE 4:** Sample demographics and sleep patterns.

	**Sleep group (*N* = 19)**		**Waking group (*N* = 20)**		***p***
			
	**Mean**	***SD***	**Mean**	***SD***	
Age	22.8	2.76	23.6	3.89	0.460
Sex ratio (M/F)	0.36	–	0.25	–	0.640
IQ	34.8	9.25	33.9	8.78	0.746
Education ratio (proportion of psychology students or psychologists)	0.37	–	0.45	–	0.604
Experience with computer games	1.53	1.31	1.10	0.97	0.253
Experience with crime riddles	1.95	1.03	1.50	0.89	0.153
Average sleep time (ST)	7:33	1:25	7:30	0:40	0.882
Average sleep onset (ST)	11:33 PM	2:33	0:18 AM	2:34	0.718
Average wake-up time (ST)	7:48 AM	1:05	7:36 AM	1:12	0.547
Sleep quality (ST)	3.95	0.70	4.15	0.67	0.364
Average sleep time (SL)	7:56	1:10	7:54	0:50	0.897
Average sleep onset (SL)	1:02 AM	1:00	0:07 AM	0:53	0.996
Average wake-up time (SL)	8:40 AM	1:11	7:58 AM	1:00	0.954
Sleep time the night previous to the experiment (SL)	7:39	1:13	8:00	1:24	0.671
Sleep onset the night before the experiment (SL)	0:53 AM	1:16	0:03 AM	1:06	0.969
Wake-up time on the day of the experiment (SL)	8:41 AM	1:09	8:02 AM	1:04	0.927

**p*-values were obtained using chi-square for the sex ratio and education ratio, and using two-sided *t*-tests for other variables. Sleep-pattern variables are expressed in hh:mm. ST, screening test; SL, sleep log.*

**TABLE 5:** Multiple regression for problem solving scores.

	**Reasonableness**	**Consistency**	**Story recall**	**Fluency**	**Flexibility**	**Originality**	**Elaboration**
**IQ**							
*β*	**0.46**	–0.14	**0.45**	**0.45**	0.28	0.06	0.23
*t*	3.12**	–0.84	3.52**	3.40**	1.80	0.37	1.56
**Sex**							
*β*	0.28	0.21	**0.45**	**0.46**	**0.40**	**0.43**	**0.59**
*t*	1.76	1.14	3.26**	3.23**	2.34*	2.51*	3.79***
**Education**							
*β*	–0.03	0.05	–0.07	–0.04	0.06	–0.03	0.17
*t*	–0.17	0.26	–0.50	–0.24	0.36	–0.16	1.03
*F*	5.36**	0.75	10.71***	9.60***	3.23*	2.97*	5.70**
*R*	0.56	0.25	0.69	0.67	0.47	0.45	0.57
*R*^2^	0.31	0.06	0.48	0.45	0.22	0.20	0.33
Adjusted *R*^2^	0.26	–0.02	0.43	0.40	0.15	0.13	0.27

Values in bold show statistically significant relationships. *p < 0.05, **p < 0.01, ***p < 0.001. *β*, standardized regression estimates.

## Discussion

In the present study, we hypothesized that, after a nap, participants would solve the problem more efficiently and more creatively than after a respective period of wakefulness. Nevertheless, the findings did not support this hypothesis. Neither quality nor creativity of the solutions generated by the participants was higher in the nap group than in the waking group. There were also no performance improvements with regard to any sleep stage or incidence of dreams. Thus, contrary to expectations, we found no effect of sleep on ill-defined problem solving. However, despite the fact that a number of studies supported sleep-related insight, reasoning, and creativity (Wagner et al., 2004; Yordanova et al., 2010, 2012; Drago et al., 2011; Ritter et al., 2012; Sio et al., 2013; Beijamini et al., 2014; Sterpenich et al., 2014; Monaghan et al., 2015; Debarnot et al., 2017), there is also accumulating evidence that sleep does not benefit problem solving (Landmann et al., 2016; Debarnot et al., 2017; Brodt et al., 2018; Schönauer et al., 2018). Our study suits this line of research.

One possible explanation of the lack of any sleep effect in our study may be associated with sleep duration. The beneficial effect of sleep on problem solving was mainly supported in studies concerning overnight sleep (Walker et al., 2002; Wagner et al., 2004; Drago et al., 2011; Ritter et al., 2012; Sio et al., 2013; Monaghan et al., 2015), while Brodt et al. (2018) and Schönauer et al. (2018) found no such effect in their nap studies, and Cai et al. (2009) reported it only for naps that included REM sleep. Furthermore, this effect is strongly related to slow-wave sleep and REM sleep (Walker et al., 2002; Cai et al., 2009; Djonlagic et al., 2009; Yordanova et al., 2010, 2012; Drago et al., 2011). There is also some evidence that dreams might provide a mechanism that enhance problem solving (Stickgold et al., 2001; Payne and Nadel, 2004; Wamsley et al., 2010a; Nieuwenhuis et al., 2013; Llewellyn, 2016; Fogel et al., 2018). In the studies with overnight sleep, participants normally obtain several cycles of both SWS and REM sleep, and usually have several dreams, while in our nap study, admittedly, all nap participants fell asleep, but only few obtained SWS and REM sleep or recalled any dreams. In addition, considering the brevity of the nap, the duration of both SWS and REM sleep in most cases did not exceed several minutes, compared with over an hour in a usual overnight study. No clear incorporations of the presented problem into the dream content were observed as well. It is possible that this amount of both SWS and REM sleep, and the lack of problem-related dream content, was not sufficient to effectively boost problem solving. Whereas there is some evidence that nap-dependent learning is comparable to that reported for an overnight sleep (Mednick et al., 2003; Lahl et al., 2008; Payne et al., 2015), this effect seems to be limited to memory consolidation. Ill-defined problem solving apparently requires either different resources not related to sleep, or longer sleep. Nevertheless, this assumption is not easy to reconcile with the results of Beijamini et al. (2014), who confirmed the sleep-related problem solving effect in a 90-min nap study with only few minutes of both SWS and REM sleep. This discrepancy might be a result of the tasks used. Both studies exploited video games; however, while Beijamini et al. (2014) used a simple logic task, we instead attempted to arrange a real-life situation and address ill-defined problem solving, using a complex and ambiguous crime riddle.

The results of our study may indicate that certain tasks profit more from sleep than others and, consequently, that certain cognitive processes benefit from sleep and others do not. As discussed by Lerner and Gluck (2019), the facilitating effects of sleep on the abstraction of hidden regularities within newly encoded stimuli strongly depend on the task specifics. Similarly, this might be the case with complex cognitive processes, i.e., the effect of sleep on problem solving might vary depending on the task used, and thus the cognitive processes involved. Whereas Beijamini et al. (2014), using a video game, demonstrated that sleep may promote the solution of problems that involve logical reasoning, no sleep-dependent improvement was observed in solving classical insight problems and magic tricks (Schönauer et al., 2018), as well as classical riddles (Brodt et al., 2018). Likewise, there is some evidence that sleep inspires insight and enhances problem solving in the NRT (Wagner et al., 2004), analogical problems (Monaghan et al., 2015), or the remote associate task (Sio et al., 2013), while Landmann et al. (2016) found no effect of sleep on creative reorganization of newly acquired memory traces in the compound remote associate task. In the present study, an interactive video game concerning a complex, elaborate crime riddle was used to address the process of solving a complex and real-world ill-defined problem. This kind of task requires much more complex cognition than simple memory reactivation that can be enhanced by sleep. In ill-defined problem solving, convergent thinking intertwines with divergent thinking, and this intertwining, called creative reasoning, may be defined as the ability to generate original, yet appropriate, solutions (Jaarsveld et al., 2010; Moreau and Engeset, 2016; Welter et al., 2017). It is largely independent from well-defined problem solving (Schraw et al., 1995; Jaarsveld et al., 2010) and requires advanced restructuring of problem representations and identifying connections, as well as reorganization and recombination of preexisting knowledge in a non-obvious way to generate new knowledge (Eysenck and Keane, 2000; Llewellyn, 2016). The results of our study do not support a notion that these processes benefit from sleep and that sleep-related memory consolidation might enhance this kind of problem restructuring and the recombination of knowledge elements necessary for ill-defined problem solving. It is possible that sleep provides only a period of brain isolation reducing interfering stimulation, which might yield comparable benefits for problem solving as a waking incubation interval (Wixted, 2004). Future studies are needed to further test if spending the incubation period asleep provides any additional improvements in solving different kinds of problems.

It is also possible that with regard to complex and ill-defined problem solving, sleep facilitates mainly the solution of personal problems. As demonstrated by Barrett (1993), problems of a personal nature were more likely to be viewed as solved after dream incubation than academic or general problems. In the present study, we used an intriguing, but rather “intellectual” problem, not related to any personal concerns. It is probable that participants did not engage in playing to such an extent as they would have if they were involved in the situation personally and that they treated the game as an intellectual pastime, admittedly interesting, attracting, and intriguing, maybe even thrilling and exciting, but not personal, and thus distantly related with their daily concerns and individually important matters. We also did not apply any task reactivation during sleep. It might be interesting to explore if a conditioned odor or auditory cue would be helpful in this kind of complex, ill-defined task. Such cues were effectively used in studies on the effect of sleep both on memory (Rihm et al., 2014) and creative performance (Ritter et al., 2012; Sterpenich et al., 2014). There is also a possibility that, in the present study, the time for the initial problem exploration was too short. In a study by Wagner et al. (2004), sleep did not enhance insight in the absence of initial training. Perhaps, in the case of a complex, ill-defined problem to solve, participants should have an opportunity of longer problem exploration. Moreover, Wamsley et al. (2010b) reported that sleep facilitated performance in navigating in a virtual maze only for participants having prior experience with navigating in a three-dimensional environment. Although we controlled for participants’ experience with crime riddles and computer games, and although there were neither any differences between the groups with regard to both variables, nor any relation to participants’ performance, we cannot rule out entirely the possible confounding effects of participants’ prior knowledge and experience. Another interesting possibility is related to the duration of the retention interval. Sleep that occurs shortly after learning is most beneficial to memory 24 h (Payne et al., 2012) or even 48–96 h after initial training (Stickgold et al., 2000). Furthermore, 30 min after learning, cramming, and napping led to similar memory improvement, but after a week, napping maintained this significant advantage, while cramming did not (Cousins et al., 2019). It is possible that considering the influence of sleep on ill-defined problem solving, also a kind of time-gap is needed for the effect to manifest. Last but not the least, the kind of video material used in the waking group might have contributed to the lack of any sleep effect. With regard to requisite task neutrality of the video material, waking participants watched a nature documentary, which was low-involving; a number of participants even assessed it as boring, and thus, they might have spent that time on pondering on the problem solution. As reported by Mooneyham and Schooler (2013), mind wandering improves creative problem solving. Therefore, in the present study, which addressed complex, ill-defined problem solving, such mind wandering might have been as helpful and beneficial as a nap. It would be noteworthy to explore the impact of various video materials in this kind of experimental situations, which might help to choose materials absorbing and task neutral at the same time.

Surprisingly, we found no differences between the groups in story recall. Because the sleep-dependent memory improvement seems to be well-established, as it was already discussed, we expected that nap participants would perform better on this measure. This effect was not observed, though. However, as already noted in *Methods*, due to the task specificity, this score was probably not exclusively a memory indicator. All the information in the game, i.e., the story plot, was presented to the participants as fragmented and disordered video clips, and the data itself were complex and ambiguous. Despite the fact that participants were given some information to memorize, acquiring that information required not only simple encoding but also prior searching and selection; thus, this measure was probably dependent not only on memory processes but also on the ability to effectively search and sort through the whole database. Furthermore, story recall was tested only once, in the end of the experimental protocol; thus the test does not allow to directly compare the effect of sleep vs. wake on memory consolidation because there was a new learning phase before the test. However, this phase was important with regard to the problem solving process—we presumed that after the nap, participants would better understand the problem situation and use better, more effective keywords that would allow to get to the most informative clips and better explore the problem and therefore would help to solve it. It was not possible to test story recall directly after the retention interval because it might have suggested some keywords, and problem solution, to the participants. Presumably, neither did this measure address pure recall, but rather the effectiveness of solving the problem, and also this result seems to be in line with our general findings, suggesting that sleep does not enhance solving ill-defined problems. Another limitation of the study is the fact that the creativity measures used in the task might be biased by the amount or quality of information that participants were able to gather (e.g., how many clips they watched or how informative the watched clips were) and probably do not capture solely creative processes. In future research, it would be noteworthy to prepare the protocol and measures in a way that would allow to discriminate different processes engaged in the problem-solving task. Perhaps, analyzing not only the final effect but also strategies of playing might bring more interesting results. In the present study, the whole course of the game and all the keywords were registered; however, our sample turned out to be too small to reasonably assess the keywords used by the participants with regard to their usefulness and importance for the problem solution. Owing to the task specificity, it was also not possible to reliably assess how informative the chosen clips were because it seems to depend not only on the content of particular clips but also on the order of watching the clips, different for each participant, and probably on individual cognitive processing as well, since each participant might have taken into account different details from the clip and might have experienced insight at different time. Some replications would be needed to allow to apply such strategy analyses and to develop alternative indicators of ill-defined problem solving.

In our study, the task performance was related only to IQ and sex. IQ was positively related to solution quality (reasonableness and story recall) and fluency. Considering sex, women had higher scores than men in all the measures of solution creativity (fluency, flexibility, originality, and elaboration) and story recall. The relationship with IQ may be easily understood, given that intelligence is a general mental capability, involving, among other things, such abilities as reasoning, planning, abstract thinking, and problem solving (Gottfredson, 1997). The relationship between solution creativity and sex is more difficult to explain, since studies concerning sex differences in creativity mostly indicate a lack of differences between men and women; on the other hand, some other studies yield mixed results (Baer and Kaufman, 2008). However, given the scarce number of male participants in the present study, this result must be treated with caution. The issue of sex differences in ill-defined problem solving and the possible confounding effects of these differences on the role of sleep in this process requires further exploration.

To summarize, based on the results of the present study, there is no evidence for any beneficial effects of sleep on ill-defined problem solving, neither with regard to quality nor creativity of the solution. With the use of a video game concerning a complex and ambiguous crime riddle, we tried to investigate how sleep affects dealing with complex, dynamic, uncertain, and open problem situations. We also made every effort to prevent effects of experimenter expectancies by precisely presenting only previously prepared, standard instructions, as well as blind scoring of both participants’ solutions and PSG recordings. Using several different measures of participants’ performance, we attempted to discriminate the effects of sleep on various cognitive processes involved in ill-defined problem solving. Presumably, sleep does not benefit any of those processes. Our study adds to a growing body of evidence that sleep probably might provide an incubation gap, but not a facilitating environment for problem solving, at least with regard to ill-defined problems. Future studies are needed to further explore the potential effects of sleep on different cognitive processes required for solving various kinds of problems.

## Data Availability Statement

The datasets generated for this study are available on request to the corresponding author.

## Ethics Statement

The present study was carried out in accordance with the recommendations of the Research Ethics Committee, Institute of Psychology, Jagiellonian University. All participants were given a letter of information and gave written informed consent before participation in accordance with the Declaration of Helsinki. They were financially compensated for their participation (or, in the pilot study, they got free lunch during the study and took part in a prize draw after the study). Study protocols were approved by the Research Ethics Committee, Institute of Psychology, Jagiellonian University.

## Author Contributions

BS, MH, MD-B, and AG conceptualized and designed the study. All authors were involved in planning. BS and MH supervised the work. AG, MD-B, DS, and MH searched the literature. MH, AG, and MD-B carried out both the pilot and the main study. MH scored PSG recordings. MH, MD-B, and DS scored participants’ solutions in the pilot study. MH, MD-B, DS, and AG performed the scoring in the main study. MH and AG processed the data and performed statistical analyses. MH drafted the manuscript and designed the tables and figures. All authors discussed the results and commented on the manuscript.

## Conflict of Interest

The authors declare that the research was conducted in the absence of any commercial or financial relationships that could be construed as a potential conflict of interest.

## Footnotes

1 www.herstorygame.com

## References

[B1] Alger S. E. Lau H. Fishbein W. (2010). Delayed onset of a daytime nap facilitates retention of declarative memory. *PLoS One* 5:e12131. 10.1371/journal.pone.0012131 20808821 PMC2924607

[B2] Antonenko D. Diekelmann S. Olsen C. Born J. Mölle M. (2013). Napping to renew learning capacity: enhanced encoding after stimulation of sleep slow oscillations. *Eur. J. Neurosci.* 37 1142–1151. 10.1111/ejn.12118 23301831

[B3] Antony J. W. Gobel E. W. O’Hare J. K. Reber P. J. Paller K. A. (2012). Cued memory reactivation during sleep influences skill learning. *Nat. Neurosci.* 15 1114–1116. 10.1038/nn.3152 22751035 PMC3498459

[B4] Baer J. Kaufman J. C. (2008). Gender differences in creativity. *J. Creat. Behav.* 42 75–105. 10.1002/j.2162-6057.2008.tb01289.x

[B5] Barrett D. (1993). The “committee of sleep”: a study of dream incubation for problem solving. *Dreaming* 3 115–122. 10.1037/h0094375

[B6] Barrett D. (2001a). Comment on baylor: a note about dreams of scientific problem solving. *Dreaming* 11 93–95. 10.1023/A:1009436621758

[B7] Barrett D. (2001b). *The Committee of Sleep: How Artists, Scientists, and Athletes use Dreams for Creative Problem-Solving – And How You Can too.* Carmarthen: Crown House Publishing Limited.

[B8] Beijamini F. Pereira S. I. R. Cini F. A. Louzada F. M. (2014). After being challenged by a video game problem, sleep increases the chance to solve It. *PLoS One* 9:e84342. 10.1371/journal.pone.0084342 24416219 PMC3885559

[B9] Berry R. B. Brooks R. Gamaldo C. E. Harding S. M. Lloyd R. M. Marcus C. L. (2015). *The AASM Manual for the Scoring of Sleep and Associated Events: Rules, Terminology and Technical Specifications, Version 2.2.* Darien, IL: American Academy of Sleep Medicine.

[B10] Brodt S. Pöhlchen D. Täumer E. Gais S. Schönauer M. (2018). Incubation, not sleep, aids problem-solving. *Sleep* 41:zsy155. 10.1093/sleep/zsy155 30113673

[B11] Cai D. J. Mednick S. A. Harrison E. M. Kanady J. C. Mednick S. C. (2009). REM, not incubation, improves creativity by priming associative networks. *Proc. Natl. Acad. Sci. U.S.A.* 106 10130–10134. 10.1073/pnas.0900271106 19506253 PMC2700890

[B12] Cartwright R. D. (1974). Problem solving: waking and dreaming. *J. Abnorm. Psychol.* 83 451–455. 10.1037/h0036811 4370286

[B13] Chambers A. M. (2017). The role of sleep in cognitive processing: focusing on memory consolidation. *WIREs Cogn. Sci.* 8:e1433 10.1002/wcs.1433 28044430

[B14] Ciechanowicz A. Jaworowska A. Matczak A. Szustrowa T. (1995). *Bateria Testów APIS-Z [APIS-Z battery].* Warszawa: Pracownia Testów PTP.

[B15] Conte F. Ficca G. (2013). Caveats on psychological models of sleep and memory: a compass in an overgrown scenario. *Sleep Med. Rev.* 17 105–121. 10.1016/j.smrv.2012.04.001 22743169

[B16] Cousins J. N. Wong K. F. Raghunath B. L. Look C. Chee M. W. L. (2019). The long-term memory benefits of a daytime nap compared with cramming. *Sleep* 42:zsy207. 10.1093/sleep/zsy207 30371902 PMC6335868

[B17] Cropley A. (2006). In praise of convergent thinking. *Creat. Res. J.* 18 391–404. 10.1207/s15326934crj1803_13

[B18] Debarnot U. Rossi M. Faraguna U. Schwartz S. Sebastiani L. (2017). Sleep does not facilitate insight in older adults. *Neurobiol. Learn. Mem.* 140 106–113. 10.1016/j.nlm.2017.02.005 28219752

[B19] Diekelmann S. Born J. (2010). The memory function of sleep. *Nat. Rev. Neurosci.* 11 114–126. 10.1038/nrn2762 20046194

[B20] Diekelmann S. Born J. Wagner U. (2010). Sleep enhances false memories depending on general memory performance. *Behav. Brain Res.* 208 425–429. 10.1016/j.bbr.2009.12.021 20035789

[B21] Diekelmann S. Landolt H.-P. Lahl O. Born J. Wagner U. (2008). Sleep loss produces false memories. *PLoS One* 3:e3512. 10.1371/journal.pone.0003512 18946511 PMC2567433

[B22] Djonlagic I. Rosenfeld A. Shohamy D. Myers C. Gluck M. Stickgold R. (2009). Sleep enhances category learning. *Learn. Mem.* 16 751–755. 10.1101/lm.1634509 19926780 PMC2788212

[B23] Dörner D. Funke J. (2017). Complex problem solving: what it is and what it is not. *Front. Psychol.* 8:1153. 10.3389/fpsyg.2017.01153 28744242 PMC5504467

[B24] Drago V. Foster P. S. Heilman K. M. Aricò D. Williamson J. Montagna P. (2011). Cyclic alternating pattern in sleep and its relationship to creativity. *Sleep Med.* 12 361–366. 10.1016/j.sleep.2010.11.009 21377416

[B25] Drummond S. P. A. Brown G. G. Gillin J. C. Stricker J. L. Wong E. C. Buxton R. B. (2000). Altered brain response to verbal learning following sleep deprivation. *Nature* 403 655–657. 10.1038/35001068 10688201

[B26] Eysenck M. W. Keane M. (2000). *Cognitive Psychology.* Philadelphia, PA: Taylor & Francis.

[B27] Fairclough S. H. Graham R. (1999). Impairment of driving performance caused by sleep deprivation or alcohol: a comparative study. *Hum. Factors* 41 118–128. 10.1518/001872099779577336 10354808

[B28] Fogel S. M. Ray L. B. Sergeeva V. De Koninck J. Owen A. M. (2018). A novel approach to dream content analysis reveals links between learning-related dream incorporation and cognitive abilities. *Front. Psychol.* 9:1398. 10.3389/fpsyg.2018.01398 30127760 PMC6088287

[B29] Frings D. (2011). The effects of group monitoring on fatigue-related Einstellung during mathematical problem solving. *J. Exp. Psychol. Appl.* 17 371–381. 10.1037/a0025131 21843017

[B30] Gais S. Born J. (2004). Declarative memory consolidation: mechanisms acting during human sleep. *Learn. Mem.* 11 679–685. 10.1101/lm.80504 15576885 PMC534696

[B31] Gottfredson L. S. (1997). Mainstream science on intelligence: an editorial with 52 signatories, history and bibliography. *Intelligence* 24 13–23. 10.1016/S0160-2896(97)90011-8

[B32] Greiff S. Wüstenberg S. Csapó B. Demetriou A. Hautamäki J. Graesser A. C. (2014). Domain-general problem solving skills and education in the 21st century. *Educ. Res. Rev.* 13 74–83. 10.1016/j.edurev.2014.10.002

[B33] Guilford J. P. (1967/1978) *Natura Inteligencji Człowieka, trans.* Czarniawska B. Kozłowski W. Radzicki J. Warszawa: PWN. [Polish translation of The nature of human intelligence].

[B34] Holm S. (1979). A simple sequentially rejective multiple test procedure. *Scand. J. Stat.* 6 65–70.

[B35] Horne J. Moseley R. (2011). Sudden early-morning awakening impairs immediate tactical planning in a changing ‘emergency’ scenario. *J. Sleep Res.* 20 275–278. 10.1111/j.1365-2869.2010.00904.x 21518064

[B36] Jaarsveld S. Lachmann T. Hamel R. van Leeuwen C. (2010). Solving and creating raven progressive matrices: reasoning in well- and ill-defined problem spaces. *Creat. Res. J.* 22 304–319. 10.1080/10400419.2010.503541

[B37] Jackson M. L. Gunzelmann G. Whitney P. Hinson J. M. Belenky G. Rabat A. (2013). Deconstructing and reconstructing cognitive performance in sleep deprivation. *Sleep Med. Rev.* 17 215–225. 10.1016/j.smrv.2012.06.007 22884948 PMC3498579

[B38] Johnston A. Gradisar M. Dohnt H. Billows M. McCappin S. (2010). Adolescent sleep and fluid intelligence performance. *Sleep Biol. Rhythms* 8 180–186. 10.1111/j.1479-8425.2010.00442.x

[B39] Jonassen D. H. (2000). Toward a design theory of problem solving. *Educ. Technol. Res. Dev.* 48 63–85. 10.1007/BF02300500

[B40] Killgore W. D. Kahn-Greene E. T. Lipizzi E. L. Newman R. A. Kamimori G. H. Balkin T. J. (2008). Sleep deprivation reduces perceived emotional intelligence and constructive thinking skills. *Sleep Med.* 9 517–526. 10.1016/j.sleep.2007.07.003 17765011

[B41] Kitchener K. S. (1983). Cognition, metacognition, and epistemic cognition: a three-level model of cognitive processing. *Hum. Dev.* 4 222–232. 10.1159/000272885

[B42] Kronholm E. Sallinen M. Suutama T. Sulkava R. Era P. Partonen T. (2009). Self-reported sleep duration and cognitive functioning in the general population. *J. Sleep Res.* 18 436–446. 10.1111/j.1365-2869.2009.00765.x 19732318

[B43] Lahl O. Wispel C. Willigens B. Pietrowsky R. (2008). An ultra short episode of sleep is sufficient to promote declarative memory performance. *J. Sleep Res.* 17 3–10. 10.1111/j.1365-2869.2008.00622.x 18275549

[B44] Landmann N. Kuhn M. Maier J. G. Feige B. Spiegelhalder K. Riemann D. (2016). Sleep strengthens but does not reorganize memory traces in a verbal creativity task. *Sleep* 39 705–713. 10.5665/sleep.5556 26518596 PMC4763358

[B45] Lau H. Alger S. E. Fishbein W. (2011). Relational memory: a daytime nap facilitates the abstraction of general concepts. *PLoS One* 6:e27139. 10.1371/journal.pone.0027139 22110606 PMC3217953

[B46] Lerner I. Gluck M. A. (2019). Sleep and the extraction of hidden regularities: a systematic review and the importance of temporal rules. *Sleep Med. Rev.* 47 39–50. 10.1016/j.smrv.2019.05.004 31252335 PMC6779511

[B47] Lewis P. A. Durrant S. J. (2011). Overlapping memory replay during sleep builds cognitive schemata. *Trends Cogn. Sci.* 15 343–351. 10.1016/j.tics.2011.06.004 21764357

[B48] Lewis P. A. Knoblich G. Poe G. (2018). How memory replay in sleep boosts creative problem-solving. *Trends Cogn. Sci.* 22 491–503. 10.1016/j.tics.2018.03.009 29776467 PMC7543772

[B49] Lim J. Dinges D. F. (2010). A meta-analysis of the impact of short-term sleep deprivation on cognitive variables. *Psychol. Bull.* 136 375–389. 10.1037/a0018883 20438143 PMC3290659

[B50] Linde L. Bergström M. (1992). The effect of one night without sleep on problem-solving and immediate recall. *Psychol. Res.* 54 127–136. 10.1007/BF00937141 1620796

[B51] Llewellyn S. (2016). Crossing the invisible line: de-differentiation of wake, sleep and dreaming may engender both creative insight and psychopathology. *Conscious Cogn.* 46 127–147. 10.1016/j.concog.2016.09.018 27718406

[B52] Llewellyn S. Hobson J. A. (2015). Not only. but also: REM sleep creates and NREM Stage 2 instantiates landmark junctions in cortical memory networks. *Neurobiol. Learn. Mem.* 122 69–87. 10.1016/j.nlm.2015.04.005 25921620

[B53] Lu W. Göder R. (2012). Does abnormal non-rapid eye movement sleep impair declarative memory consolidation? Disturbed thalamic functions in sleep and memory processing. *Sleep Med. Rev.* 16 389–394. 10.1016/j.smrv.2011.08.001 21889375

[B54] Mednick S. Nakayama K. Cantero J. L. Atienza M. Levin A. A. Pathak N. (2002). The restorative effect of naps on perceptual deterioration. *Nat. Neurosci.* 5 677–681. 10.1038/nn864 12032542

[B55] Mednick S. Nakayama K. Stickgold R. (2003). Sleep-dependent learning: a nap is as good as a night. *Nat. Neurosci.* 6 697–698. 10.1038/nn1078 12819785

[B56] Monaghan P. Sio U. Lau S. Woo H. Linkenauger S. Ormerod T. (2015). Sleep promotes analogical transfer in problem solving. *Cognition* 143 25–30. 10.1016/j.cognition.2015.06.005 26113445

[B57] Mooneyham B. W. Schooler J. W. (2013). The costs and benefits of mind-wandering: a review. *Can. J. Exp. Psychol.* 67 11–18. 10.1037/a0031569 23458547

[B58] Moreau C. P. Engeset M. G. (2016). The downstream consequences of problem-solving mindsets: how playing with LEGO influences creativity. *J. Market. Res.* 53 18–30. 10.1509/jmr.13.0499

[B59] Nebes R. D. Buysse D. J. Halligan E. M. Houck P. R. Monk T. H. (2009). Self-reported sleep quality predicts poor cognitive performance in healthy older adults. *J. Gerontol. B Psychol. Sci. Soc. Sci.* 64 180–187. 10.1093/geronb/gbn037 19204069 PMC2655169

[B60] Newell A. Simon H. A. (1972). *Human Problem Solving.* Englewood Cliffs, NJ: Prentice Hall.

[B61] Nieuwenhuis I. L. C. Folia V. Forkstam C. Jensen O. Petersson K. M. (2013). Sleep promotes the extraction of grammatical rules. *PLoS One* 8:e65046. 10.1371/journal.pone.0065046 23755173 PMC3673983

[B62] Pagel J. F. Kwiatkowski C. Broyles K. E. (1999). Dream use in film making. *Dreaming* 9 247–256. 10.1023/A:1021384019464

[B63] Payne J. D. Kensinger E. A. Wamsley E. J. Spreng R. N. Alger S. E. Gibler K. (2015). Napping and the selective consolidation of negative aspects of scenes. *Emotion* 15 176–186. 10.1037/a0038683 25706830 PMC5846328

[B64] Payne J. D. Nadel L. (2004). Sleep, dreams, and memory consolidation: the role of the stress hormone cortisol. *Learn. Mem.* 11 671–678. 10.1101/lm.77104 15576884 PMC534695

[B65] Payne J. D. Tucker M. A. Ellenbogen J. M. Wamsley E. J. Walker M. P. Schacter D. L. (2012). Memory for semantically related and unrelated declarative information: the benefit of sleep, the cost of wake. *PLoS One* 7:e33079. 10.1371/journal.pone.0033079 22457736 PMC3310860

[B66] Perdomo V. L. Hofman W. F. Talamini L. M. (2018). Sleep fosters insight into real-life problems. *Arch. Ital. Biol.* 156 87–98. 10.12871/00039829201831 30324605

[B67] Plessow F. Kiesel A. Petzold A. Kirschbaum C. (2011). Chronic sleep curtailment impairs the flexible implementation of task goals in new parents. *J. Sleep Res.* 20 279–287. 10.1111/j.1365-2869.2010.00878.x 20704644

[B68] Przybylski A. K. Rigby C. S. Ryan R. M. (2010). A motivational model of video game engagement. *Rev. Gen. Psychol.* 14 154–166. 10.1037/a0019440

[B69] Rasch B. Born J. (2013). About sleep’s role in memory. *Physiol. Rev.* 93 681–766. 10.1152/physrev.00032.2012 23589831 PMC3768102

[B70] Reitman W. J. (1965). *Cognition and Thought: An Information Processing Approach.* Oxford: John Wiley & Sons.

[B71] Rihm J. S. Diekelmann S. Born J. Rasch B. (2014). Reactivating memories during sleep by odors: odor specificity and associated changes in sleep oscillations. *J. Cogn. Neurosci.* 26 1806–1818. 10.1162/jocn_a_00579 24456392

[B72] Ritter S. M. Strick M. Bos M. W. Van Baaren R. B. Dijksterhuis A. (2012). Good morning creativity: task reactivation during sleep enhances beneficial effect of sleep on creative performance. *J. Sleep Res.* 21 643–647. 10.1111/j.1365-2869.2012.01006.x 22404078

[B73] Schönauer M. Brodt S. Pöhlchen D. Breßmer A. Danek A. Gais S. (2018). Sleep does not promote solving classical insight problems and magic tricks. *Front. Hum. Neurosci.* 12:72 10.3389/fnhum.2018.00072 PMC5834438 29535620

[B74] Schraw G. Dunkle M. E. Bendixen L. D. (1995). Cognitive processes in well-defined and ill-defined problem solving. *Appl. Cogn. Psychol.* 9 523–538. 10.1002/acp.2350090605

[B75] Schredl M. Erlacher D. (2007). Self-reported effects of dreams on waking-life creativity: an empirical study. *J. Psychol.* 141 35–46. 10.3200/JRLP.141.1.35-46 17312685

[B76] Shute V. Wang L. Greiff S. Zhao W. Moore G. (2016). Measuring problem solving skills via stealth assessment in an engaging video game. *Comp. Hum. Behav.* 63 106–117. 10.1016/j.chb.2016.05.047

[B77] Simon H. A. (1986). *Report of the Research Briefing Panel on Decision Making and Problem Solving.* Washington, DC: National Academy of Sciences.

[B78] Sio U. N. Monaghan P. Ormerod T. (2013). Sleep on it, but only if it is difficult: effects of sleep on problem solving. *Mem. Cogn.* 41 159–166. 10.3758/s13421-012-0256-7 23055117

[B79] Sterpenich V. Schmidt C. Albouy G. Matarazzo L. Vanhaudenhuyse A. Boveroux P. (2014). Memory reactivation during rapid eye movement sleep promotes its generalization and integration in cortical stores. *Sleep* 37 1061–1075. 10.5665/sleep.3762 24882901 PMC4015380

[B80] Stickgold R. Hobson J. A. Fosse R. Fosse M. (2001). Sleep, learning, and dreams: off-line memory reprocessing. *Science* 294 1052–1057. 10.1126/science.1063530 11691983

[B81] Stickgold R. James L. Hobson J. A. (2000). Visual discrimination learning requires sleep after training. *Nat. Neurosci.* 3 1237–1238. 10.1038/81756 11100141

[B82] Stickgold R. Walker M. P. (2005). Memory consolidation and reconsolidation: what is the role of sleep? *Trends Neurosci.* 28 408–415. 10.1016/j.tins.2005.06.004 15979164

[B83] Torrance E. P. (1974). *Torrance Tests of Creative Thinking.* Bensenville, IL: Scholastic Testing Services.

[B84] Uga V. Lemut M. C. Zampi C. Zilli I. Salzarulo P. (2006). Music in dreams. *Conscious Cogn.* 15 351–357. 10.1016/j.concog.2005.09.003 16243543

[B85] Wagner U. Gais S. Haider H. Verleger R. Born J. (2004). Sleep inspires insight. *Nature* 427 352–355. 10.1038/nature02223 14737168

[B86] Walker M. P. Brakefield T. Hobson J. A. Stickgold R. (2003). Dissociable stages of human memory consolidation and reconsolidation. *Nature* 425 616–620. 10.1038/nature01930 14534587

[B87] Walker M. P. Liston C. Hobson J. A. Stickgold R. (2002). Cognitive flexibility across the sleep-wake cycle: REM-sleep enhancement of anagram problem solving. *Cogn. Brain Res.* 14 317–324. 10.1016/S0926-6410(02)00134-9 12421655

[B88] Wamsley E. J. Tucker M. Payne J. D. Benavides J. Stickgold R. (2010a). Dreaming of a learning task is associated with enhanced sleep-dependent memory consolidation. *Curr. Biol.* 20 850–855. 10.1016/j.cub.2010.03.027 20417102 PMC2869395

[B89] Wamsley E. J. Tucker M. Payne J. D. Stickgold R. (2010b). A brief nap is beneficial for human route-learning: the role of navigation experience and EEG spectral power. *Learn. Mem.* 17 332–336. 10.1101/lm.1828310 20581255 PMC2904102

[B90] Welter M. M. Jaarsveld S. Lachmann T. (2017). Problem space matters: the development of creativity and intelligence in primary school children. *Creat. Res. J.* 29 125–132. 10.1080/10400419.2017.1302769 29740367

[B91] Williamson A. M. Feyer A.-M. (2000). Moderate sleep deprivation produces impairments in cognitive and motor performance equivalent to legally prescribed levels of alcohol intoxication. *Occup. Environ. Med.* 57 649–655. 10.1136/oem.57.10.649 10984335 PMC1739867

[B92] Wixted J. T. (2004). The psychology and neuroscience of forgetting. *Annu. Rev. Psychol.* 55 235–269. 10.1146/annurev.psych.55.090902.141555 14744216

[B93] Wouters P. Nimwegen C. van der Spek E. (2013). A meta-analysis of the cognitive and motivational effects of serious games. *J. Educ. Psychol.* 2 249–265. 10.1037/a0031311

[B94] Xu L. Jiang C. Q. Lam T. H. Liu B. Jin Y. L. Zhu T. (2011). Short or long sleep duration is associated with memory impairment in older Chinese: the Guangzhou biobank cohort study. *Sleep* 34 575–580. 10.1093/sleep/34.5.575 21532950 PMC3079936

[B95] Yordanova J. Kolev V. Wagner U. Born J. Verleger R. (2012). Increased Alpha (8–12 Hz) activity during slow wave sleep as a marker for the transition from implicit knowledge to explicit insight. *J. Cogn. Neurosci.* 24 119–132. 10.1162/jocn_a_00097 21812555

[B96] Yordanova J. Kolev V. Wagner U. Verleger R. (2010). Differential associations of early- and late-night sleep with functional brain states promoting insight to abstract task regularity. *PLoS One* 5:e9442. 10.1371/journal.pone.0009442 20195475 PMC2829083

