# Supplementary material for: Ill-Defined Problem Solving Does Not Benefit From Daytime Napping
Source: Front Psychol. 2020 Apr 9;11:559. doi: 10.3389/fpsyg.2020.00559 (PMC7161088; doi:10.3389/fpsyg.2020.00559)
Supplement: Supplementary file 1 [file Data_Sheet_1.pdf]

Supplementary Material

Ill-defined problem solving does not benefit from daytime napping

Małgorzata Hołda, Anna Głodek, Malwina Dankiewicz-Berger, Dagna Skrzypińska, Barbara Szmigielska

*** Correspondence:** Małgorzata Hołda: m.holda@uj.edu.pl

1. Pilot study

To test the feasibility of methods and procedures, a pilot study was conducted first, then some corrections were applied to the main study protocol and research tools. Subsequently the main study was conducted. Due to the changes introduced to the study procedure it was not possible to include the results of the pilot study in the final analyses. However, some preliminary analyses of the mere pilot study findings were also conducted. They are presented here together with more detailed description of the methods, followed by the modifications of the study protocol implemented after the pilot study.

1.1 Methods

1.1.1 Participants

Twenty participants took part in the pilot study, 13 females and 7 males, aged 22-25, students of various programmes (10 psychology students). They were recruited by internet advertisements. The inclusion criterion was ability to sleep during the day. The participants were randomly assigned to two experimental conditions: sleep group (5 females and 5 males) and waking group (8 females, 2 males). They got free lunch during the study and took part in a prize draw after the study.

1.1.2 Procedure and measures

Participants were asked to keep sleep logs for a week prior to the study in order to monitor their sleep-wake cycle. On the experimental day they were informed in detail about all the experimental procedures, study goals, and instructions, they were also interviewed on basic demographics, as well as experience with computer games and with crime stories or riddles (books, films, etc.).

The experimental protocol in the pilot study was generally parallel to the main study protocol. It comprised three stages: problem presentation, retention interval, and testing stage. The problem was presented to the participants in the form of an interactive computer game, concerning a complex, elaborate crime riddle, with no definitive solution (for more detailed game description see the main

paper). Participants played the game for 60 minutes. During the retention interval the participants – depending on the condition – took a 90 minutes nap or stayed awake. Sleep participants underwent polysomnography recording, performed in accordance with standardized techniques, using a 15-channel polysomnographic montage, which included EEG, EOG, and chin EMG channels (for more detailed description see the main paper). Waking participants performed activities not related to the experimental problem (they watched science fiction videos).

In the testing stage all participants played the game for another 60 minutes and then took the final test, comprising 37 questions concerning the presented problem. Time for filling out the test was not limited, it usually took approximately 15-20 minutes. The solutions generated by the participants were assessed for their reasonableness, consistency, and story recall, as well as fluency, flexibility, originality, and elaboration (for more detailed description of the scales see the main paper). All the assessments were performed by a panel of three independent expert raters, blind to participants' group assignment.

1.2 Results

Due to the changes introduced to the study protocol it was not possible to include the results of the pilot study in the final analyses. Therefore, some preliminary analyses of the mere pilot study findings were conducted.

In order to test the agreement among the three raters who assessed participants' solutions, Kendall's coefficient of concordance was calculated. The obtained coefficients were high for reasonableness, story recall, fluency, flexibility, originality, and elaboration, and lower, but still acceptable, for consistency (see Table 1). Therefore, the raters' scores were averaged and those aggregated scores were used in further analyses.

Table 1. Coefficients of concordance among three raters' scores of participants' solutions (pilot study)

	Kendall's *W*
Reasonableness	0.96
Consistency	0.44
Story recall	0.96
Fluency	0.96
Flexibility	0.86
Originality	0.85
Elaboration	0.98

In order to compare the performance of the sleep and waking groups independent *t*-tests were used, adjusted for multiple comparisons with the sequentially rejective multiple-test procedure (Bonferroni-Holm correction; Holm, 1979). None of the effects was significant. The results are presented in Table 2, and Figure 1 shows box-and-whisker plots for all the effects. There was also no effect with regard to any sleep stage or incidence of dreams.

Table 2. Effects of sleep on problem solving (pilot study)

	Sleep group (N=10)		Waking group (N=10)		$t(18)$	p
	Mean	*SD*	Mean	*SD*		
Reasonableness	22.6	5.24	21.8	7.47	- 0.277	0.785
Consistency	0.99	0.005	0.99	0.015	- 0.908	0.376
Story recall	29.6	3.91	31.6	4.88	0.994	0.333
Fluency	28.0	4.70	30.7	4.40	1.360	0.191
Flexibility	8.7	3.54	10.3	2.88	1.063	0.302
Originality	1.5	1.15	1.8	1.07	0.605	0.552
Elaboration	39.7	7.93	45.7	6.21	1.894	0.074

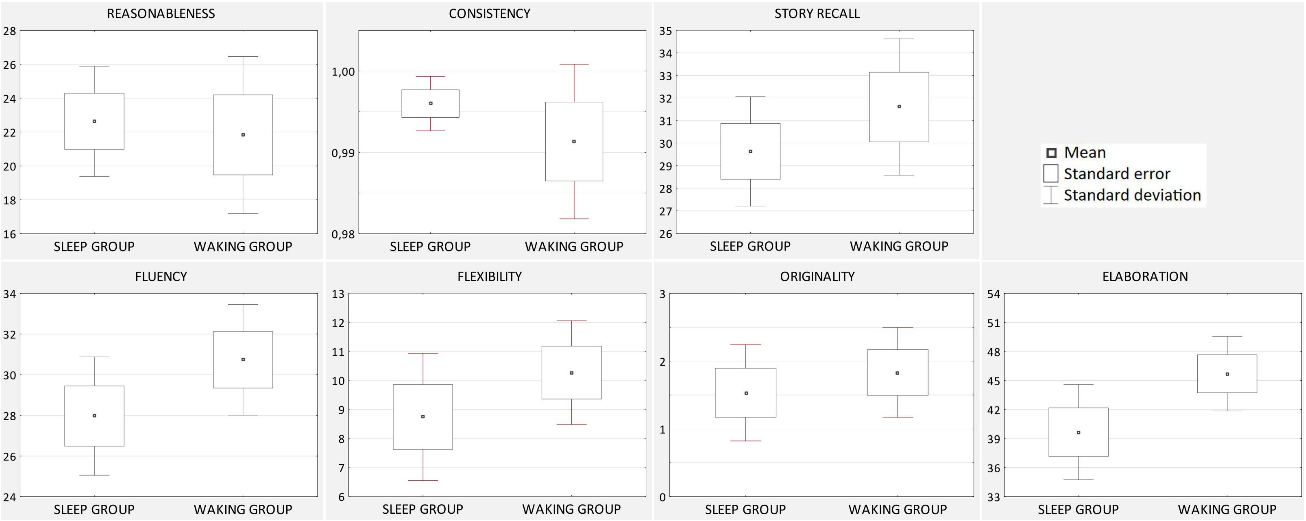

Figure 1. Effects of sleep on problem solving. Differences between sleep and waking group in task performance (means, standard errors, and standard deviations; pilot study).

Sleep and waking groups were balanced for age (*t*=1.14, *p*=0.27) and sex (χ2=1.98, *p*=0.16); however, in the waking group there were significantly more participants who were studying psychology (8 in the waking group vs. 2 in the sleep group; χ^2=7.20, $p<0.01$). There was no difference between groups in experience with games or crime riddles.

2 Protocol modifications

The main goal of the pilot study was to test the feasibility of methods and procedures used, therefore, some necessary corrections were subsequently applied to the main study protocol and measures. Changes that we decided to introduce included: changing the video film the waking group watched during the retention interval, shortening the time of playing the game after the retention interval, and revising the final test.

In the pilot study the waking group watched science fiction videos not related to the experimental problem, which were supposed to intrigue participants and attract their attention so as to prevent

them from working on the problem solution. However, it turned out that this was accomplished in excess - some participants said they would prefer to continue watching videos than to continue the game and they returned to the game reluctantly. It was also not certain if the material was really not related to the task and if it could not induce some ideas that might help participants in solving the problem. Mindful of participants' motivation and actual neutrality of the video material, in the main study we decided to use a nature documentary instead.

Moreover, the time of playing the game after the retention interval was shortened in the main study from 60 to 40 minutes. This change was made due to the fact that a number of participants after approximately 30-40 minutes of playing reported finishing the game, despite the fact they could continue playing - but the game doesn't have an explicit, definite ending, so they just presumed at a moment that there was no possibility to continue. To avoid this kind of situations, which might disrupt participants' motivation or cause differentiation in protocol timing, we decided to shorten this stage of the experiment. Moreover, some additional playing trials revealed that this time is sufficient to explore the story plot and the problem presented in the game.

Last but not least, the final test was revised to allow obtaining more elaborate and detailed responses, which might more clearly reveal participants' reasoning process. Vague or suggesting questions were modified or removed, some questions were added or expanded, and all the multiple choice questions (6) were replaced with open questions to obtain more detailed responses. New scoring rules were also prepared.
